# Small Molecule EGFR Inhibitors as Anti-Cancer Agents: Discovery, Mechanisms of Action, and Opportunities

**DOI:** 10.3390/ijms24032651

**Published:** 2023-01-31

**Authors:** Tanzida Zubair, Debasish Bandyopadhyay

**Affiliations:** 1 Department of Chemistry, The University of Texas Rio Grande Valley, 1201 West University Drive, Edinburg, TX 78539, USA; 2 School of Earth Environment & Marine Sciences (SEEMS), The University of Texas Rio Grande Valley, 1201 West University Drive, Edinburg, TX 78539, USA

**Keywords:** epidermal growth factor receptor (EGFR), cancer therapeutics, small molecule inhibitors, anticancer drugs, natural cancer drugs, heterocycles, polycyclic compounds

## Abstract

Epidermal growth factor receptors (EGFRs) are a class of receptor tyrosine kinase that are also called ErbB1 and HER1. EGFR tyrosine kinase activity inhibition is considered a promising therapeutic strategy for the treatment of cancer. Many small-molecule inhibitors of EGFR tyrosine kinase (EGFR-TK), from medicinally privileged molecules to commercial drugs, have been overviewed. Particular attention has been paid to the structure of the molecule and its mechanism of action if reported. Subsequent classification of the molecules under discussion has been carried out. Both natural and synthetic and reversible and irreversible EGFR-tyrosine kinase inhibitors have been discussed. Various types of cancers that are caused by overexpression of the EGFR gene, their possible molecular origins, and their natures have also been counted in this article. Because the EGFR signaling pathway controls the proliferation, growth, survival, and differentiation of cells, and the mutated EGFR gene overproduces EGFR protein, which ultimately causes several types of cancer, proper understanding of the molecular dynamics between the protein structure and its inhibitors will lead to more effective and selective EGFR-TKIs, which in turn will be able to save more lives in the battle against cancer.

## 1. Introduction

Epidermal growth factor receptor (EGFR) inhibitors are a class of drugs that are used to treat several common malignancies, including breast, colon, lung, and pancreatic cancer [1]. Epidermal growth factor receptor (EGFR) has four different members, with similar structural features, such as EGFR (HER1/ErbB1), ErbB2 (HER2/neu), ErbB3 (HER3), and ErbB4 (HER4). The action of EGFR is a key mediator in the cell signaling pathways involving cell proliferation, apoptosis, angiogenesis, and metastatic spread [2]. Two classes of anti-EGFR drugs are currently on the market. One of these is small-molecule tyrosine kinase inhibitors (TKIs), which can bind to the intracellular catalytic site of the EGFR, and another is monoclonal antibodies (mAbs) against the extracellular domain that can block the dimerization of the receptor. The first EGFR-TKIs to be approved by the FDA for the treatment of advanced non-small-cell lung cancer were gefitinib, afatinib, dacomitinib, erlotinib, and osimertinib. Erlotinib, lapatinib, and icotinib bind to the EGFR in a reversible manner and have been approved as first-line therapies for advanced NSCLC and pancreatic cancer [3]. There has been a broad study on the EGFR family regarding its general role in signal transduction as well as in the pathogenesis of a variety of malignancies such as lung, breast, stomach, colorectal, head and neck, and pancreatic carcinomas. Lapatinib and neratinib are approved for the treatment of HER2-positive breast cancer, which targets ErbB2. Worldwide, the number of newly diagnosed lung cancers is estimated at 2.09 million, and the number of deaths is estimated at about 1.76 million. The total number of newly diagnosed breast cancers in women worldwide is estimated at 2.08 million, and the number of deaths is estimated to be 627,000. Approximately 20% of newly diagnosed breast cancers are due to the overexpression of ErbB2 [4].

## 2. EGFR Family

EGFR has been identified as a biomolecular target for cancer since its discovery. The EGFR is a 170 kDa molecular weight transmembrane glycoprotein with a potential glycosylation site at the N-terminal [5]. The EGFR gene is active in many malignancies, but it is especially high in brain and head and neck tumors. For improving cancer treatment, the researcher’s leading target is the EGFR family. The synonyms and discovery of four members of the EGFR family: EGFR, ErbB2, ErbB3, and ErbB4 are shown in Table 1.

The EGFR family members control the production, survival, movement, and structure of many embryonic and adult cell types. Ligand-dependent oligomerization regulates signaling from EGFR, ErbB3, and ErbB4, but oligomerization with ligated forms of the other family members regulates ErbB2. Three members of the family (EGFR, ErbB2, and ErbB4) signal through active tyrosine kinase scaffolds, whereas the fourth, ErbB3, signals by ligand-induced oligomerization and activation of the other members of the family. The majority of ligand-activated EGFR signaling pathway research is carried out on cell lines. It is crucial to understand that mutations will activate the signaling systems in most of these cell lines. Signaling studies are typically complex, making it difficult to determine which connections are important for normal regulatory processes and which are a result of the cells “transformed” condition. However, if we properly understand the responses of these pathways to EGF ligands in both normal and cancerous cells, we will be in a much better position to select the best treatment combination for cancer [6].

## 3. EGFR in Cancer Cell Mechanism

The EGFR pathway was first explored when Stanley Cohen discovered EGF in 1963 and the EGFR gene later in the 1980s. The EGFR tyrosine kinase family has been linked to the development and progression of several malignancies. Type I receptor tyrosine kinases, or ErbB, are another name for this family of EGFRs. The receptors are inert as a single unit (monomer), but when bound by a ligand, they form active pairs (dimers). The internal tyrosine kinase domain is activated by dimer formation, which catalyzes protein phosphorylation. The signal eventually makes its way into the nucleus, where it is recognized by cyclins (particularly cyclin D) and cyclin-dependent kinases, resulting in cell division activation. As a result, non-physiological EGFR activation can result in unregulated cell division and, eventually, a growing tumor mass. However, activation of EGFR results in the activation of other intracellular pathways, including prevention of apoptosis, stimulation of angiogenesis, and promotion of metastases, in addition to the intracellular activation of the RAS pathway, which leads to cell proliferation. Because EGFR is frequently overexpressed in human tumors, there is a compelling reason to try to inhibit it in order to improve cancer treatment [7]. Due to EGFR gene amplification and/or protein overexpression, mutations, or in-frame deletions, EGFR signaling is commonly changed in a variety of human malignancies. Glioblastoma and lung cancer mutations that are resistant to antibody-mediated EGFR inhibition and mutations were observed in colorectal malignancies [8]. HER2 overexpression is found in about 30% of breast cancer. Human cancer has been associated with increased or decreased expression of ErbB3 or ErbB4. In breast cancer, ErbB3 expression appears to promote tumor growth, whereas ErbB4 expression appears to perform as a weak tumor suppressor. The data analysis revealed ten significant cancer types that both overexpress EGFR in comparison to normal tissues and have been thoroughly examined to allow assessments of the association between EGFR and cancer prognosis [9].

## 4. Classification of the Approved Small Molecule EGFR Inhibitors

### 4.1. Based on the Mode of Inhibition of the EGFR Kinase Activity

Tyrosine kinase inhibitors (TKIs) are small molecules that inhibit the signaling pathway by inhibiting tyrosine *trans*-phosphorylation, which inhibits ligand-induced EGFR activation. The approved EGFR-TKIs can be classified as reversible or irreversible inhibitors depending on how they inhibit EGFR kinase activity (Table 2) [10]. Reversible inhibitors compete for the ATP binding site in the EGFR through noncovalent interactions such as electrostatic, hydrogen-bonding, and hydrophobic interactions [10]. The majority of molecules currently being developed compete with the ATP-binding site and have high selectivity for EGFR tyrosine kinase; the majority are reversible inhibitors, while a few irreversible inhibitors have recently been synthesized [11]. FDA-approved EGFR/ErbB1 inhibitors such as afatinib, dacomitinib, erlotinib, gefitinib, and osimertinib have been developed for the treatment of non-small cell lung cancer (NSCLC). Lapatinib and neratinib are ErbB2-targeting drugs that have been approved to treat HER2-positive breast cancer. Early research found that about 10% of patients with NSCLC had extremely good responses to gefitinib. The FDA approved gefitinib for the treatment of NSCLC in 2003 and erlotinib in 2004 [4]. 

### 4.2. Classification Based on the Target Kinases

The approved EGFR-TKIs can also be divided into three categories based on their target kinases (Table 3): The first is selective EGFR inhibitors such as gefitinib, which targets EGFR with high selectivity. The second category is dual EGFR inhibitors such as lapatinib, which target EGFR and ErbB2. The third is multi-kinase inhibitors such as brigatinib, pyrotinib, and vandetanib [10].

### 4.3. Classification into First-, Second-, Third-, and Fourth-Generation TKIs

#### 4.3.1. First-Generation EGFR-TKIs

In 2003 and 2004, the US Food and Drug Administration (FDA) approved quinazoline-based derivatives such as gefitinib and erlotinib from the first-generation EGFR-TKIs for the treatment of NSCLC patients [12].

#### 4.3.2. Gefitinib

**Figure 1:**
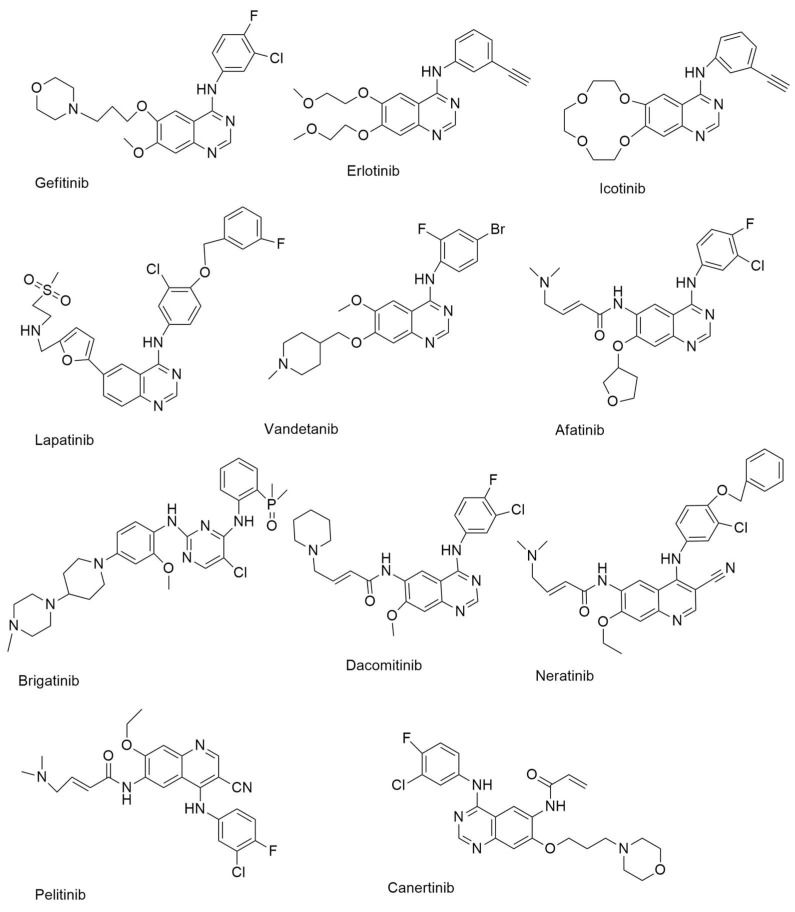
First-generation and second-generation small molecule EGFR-TKIs.

Gefitinib (Figure 1) is a TKI that is used to treat individuals with metastatic NSCLC who have particular EGFR mutations in their tumors [13]. It was authorized on 13 July 2015, for metastatic EGFR mutation-positive NSCLC [14]. At low nanomolar concentrations, gefitinib showed substantial anti-proliferative effects in A549 NSCLC, MCF-7 breast cancer, HX62 ovarian, prostrate, and colorectal carcinoma human xenograft models. In the PDB, gefitinib is found as a co-crystallized ligand in eight crystal forms. Gefitinib, in combination with the EGFR kinase domain, is seen in these crystals.

#### 4.3.3. Erlotinib

On 18 November 2004, the FDA approved erlotinib (Figure 1) for the treatment of locally progressed or metastatic NSCLC, and it was then also approved as a first-line treatment for pancreatic cancer patients in conjunction with gemcitabine [13]. Erlotinib, like gefitinib, works as an ATP analog, competing with ATP binding sites inside RTKs to inhibit proliferation, cell cycle arrest, and apoptosis [15].

#### 4.3.4. Icotinib

Icotinib (Figure 1) is a highly selective EGFR-TKI that was approved for use in the treatment of NSCLC in China in June 2018. Icotinib’s anticancer efficacy was also studied in A549 lung cancer cells. The results showed that icotinib had an IC_50_ of 8.8 µM [16].

#### 4.3.5. Lapatinib

Lapatinib (Figure 1) is an orally active, reversible, and selective RTK inhibitor that targets both EGFR and HER2. Lapatinib is a dual EGFR/ErbB2 kinase inhibitor that was initially approved by the FDA in 2007 for advanced or metastatic breast cancer in combination with capecitabine [17]. It was also discovered to have an action against AKT overexpressing human tumor xenografts [15]. The inhibitory activity of lapatinib against pure EGFR, ErbB2, and ErbB4 was investigated by Rusnak et al. With IC_50_ values of 9.8 and 10.2 nM, respectively, the results demonstrated significant inhibitory actions against EGFR and ErbB2 [18]. Lapatinib also inhibited the growth of gastric cancer cells [19].

#### 4.3.6. Vandetanib

Vandetanib (Figure 1) inhibits angiogenesis and cancer cell proliferation by inhibiting several kinases [20]. On 6 April 2011, the FDA approved it for use in the treatment of patients with thyroid cancer [21]. It suppressed EGFR with an IC_50_ of 0.5 µM and inhibited oncogenic RETK (REarranged during Transfection Kinase) activity with an IC_50_ value of 0.13 µM [10].

### 4.4. Second-Generation EGFR-TKIs

EGFR TKIs of the second generation, including afatinib, dacomitinib, neratinib, canertinib, and pelitinib, were developed to treat T790M mutations.

As second-generation EGFR TKIs, several 4-anilinoquinazoline and 4-anilinoquinoline compounds have been synthesized and tested. Targeting other human epidermal growth factor receptors (HER-family members such as HER2) and dose-limiting toxicity caused by targeting both mutant T790M and wild-type EGFR are two important limitations for the continued clinical development of these drugs [22].

#### 4.4.1. Afatinib

Afatinib (Figure 1) is an anilinoquinazoline-based irreversible EGFR inhibitor [23]. The FDA authorized afatinib for treating patients with metastatic NSCLC who had an EGFR mutation (EGFR exon 19 deletions or exon 21 (L858R) replacement mutations) [24]. Afatinib is a selective inhibitor of wild-type EGFR, HER2, and HER4 enzymatic activity, with good outcomes against single and double mutant EGFR with an IC_50_ of 10 nM [25]. In the protein data bank, afatinib was identified as a bound ligand in three crystal structures. The crystal structure of afatinib coupled to the EGFR kinase. Afatinib, in addition to these reversible binding interactions, has been demonstrated to inhibit ErbB receptor family members irreversibly. This inhibition is dependent on afatinib’s capacity to establish a covalent bond with cysteine residues in EGFR, HER2, and HER4, resulting in tyrosine kinase activity suppression [10].

#### 4.4.2. Brigatinib

Brigatinib (Figure 1) is a multi-kinase inhibitory phosphorous derivative. The FDA authorized it for use in patients with ALK-positive NSCLC on 28 April 2017 [10]. Brigatinib targets ALK, ROS1, FLT3, and mutant EGFR [26]. The EGFR, ALK, FLT3, and other kinases are also inhibited by brigatinib. This drug is generally prescribed for the treatment of non-small cell lung cancer (NSCLC), anaplastic large cell lymphoma (ALCL), and neuroblastomas [27].

#### 4.4.3. Dacomitinib

Dacomitinib is defined as a pan-HER inhibitor that is taken once daily [28]. The FDA authorized dacomitinib (Figure 1) for treating metastatic NSCLC with EGFR exon 19 deletion or exon 21 L858R substitution mutations. Dacomitinib’s kinase inhibitory action was tested against the HER family’s wild type [29]. Another irreversible EGFR, HER2, and HER4 inhibitor, dacomitinib had IC_50_ values of 6.0, 45.7, and 73.7 nM. in enzymatic assays, respectively. The protein data bank has two crystal structures for dacomitinib. Dacomitinib, in combination with the wild-type EGFR kinase domain (PDB ID: 4I23) and the T790M EGFR kinase domain are included in these protein crystals (PDF ID: 4I24) [10].

#### 4.4.4. Neratinib

Neratinib (Figure 1) is an irreversible EGFR, HER2/4 receptor tyrosine kinase inhibitor that was approved for use in the treatment of HER2-positive breast cancer on 17 July 2017 [10]. It was approved for HER2-positive metastatic breast cancer in 2020 [30]. In both enzymatic (IC_50_ values of 92 and 59 nm., respectively) and cell-based studies, neratinib is an irreversible inhibitor of EGFR and HER2 (it suppressed the growth of NCI-H1975 cells) [31].

#### 4.4.5. Pelitinib

Pelitinib (Figure 1) is a strong inhibitor of EGFR kinase enzymatic activity with an IC_50_ value of 39 nM. It is a selective and irreversible EGFR inhibitor [32].

#### 4.4.6. Canertinib

Canertinib (Figure 1) is 3-chloro 4-fluoro 4-anilinoquinazoline. It is an irreversible low-molecular-weight pan-EGFR family TKI that can be taken orally and is a new generation TKI that works by alkylating a cysteine residue found only in ErbB family receptors, leading in permanent blockage of these receptors and their downstream mitogenic signaling pathways [15]. With IC_50_ values of 0.8, 19.0, and 7.0 nM., respectively, canertinib inhibited the enzymatic activities of EGFR, HER2, and HER4 irreversibly [33]

### 4.5. Third-Generation EGFR-TKIs

The first third-generation EGFR-TKIs with good selectivity over double mutant EGFR is WZ4002 8, which has a 4-aminopyrimidine core structure [34].

#### 4.5.1. Osimertinib

**Figure 2:**
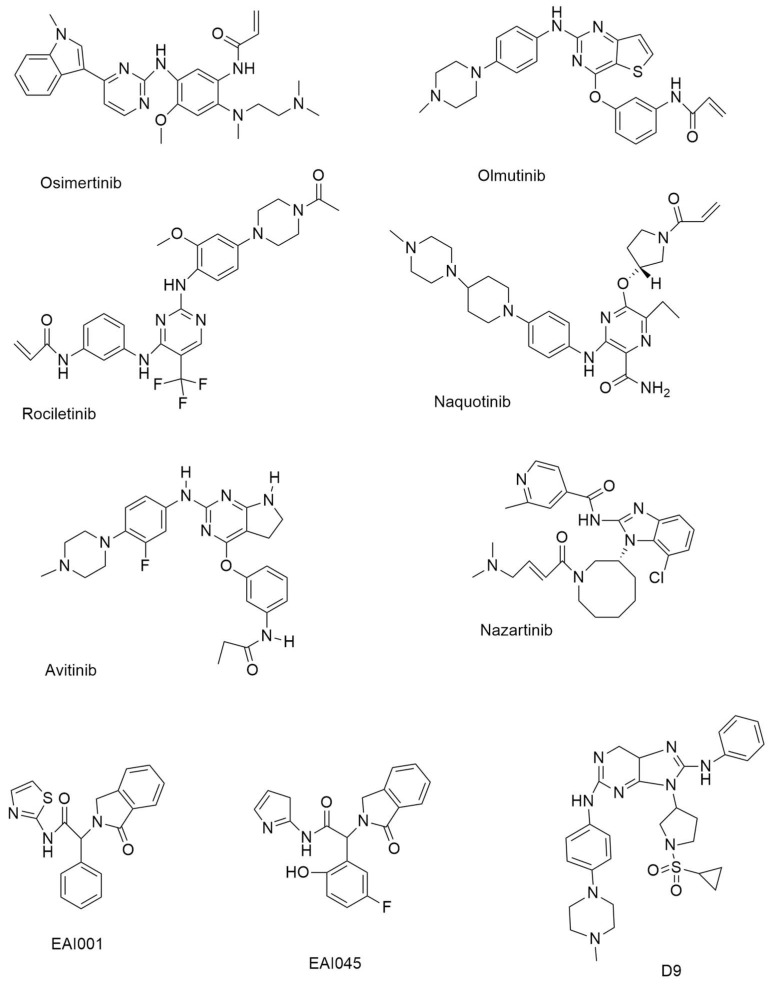
Third-generation and Fourth-generation small molecule EGFR-TKIs.

On 13 November 2015, the FDA certified Osimertinib (Figure 2) as a third-generation, irreversible EGFR-TKI for the treatment of EGFR T790M mutation-positive NSCLC [35]. Osimertinib’s inhibitory efficacy against wild-type and mutant EGFR variants was studied [25]. With an IC_50_ of 17 and 15 nM, respectively, Osimertinib inhibits the growth of PC-9 cells and H1975 cells (both cell types belong to NSCLC). Osimertinib is the only third-generation EGFR TKI currently on the market, and it has been noted that some medicines may interact with it [36].

#### 4.5.2. Olmutinib

Olmutinib is a thieno [3,2-*d*] pyrimidine-based drug, inhibiting H1975 and HCC827 cells with GI_50_ values of 9.2 and 10 nM., respectively. Olmutinib (Figure 2) is a third-generation EGFR-TKI that was approved in South Korea in May 2016 for the treatment of patients with NSCLC who have the T790M mutation [37]. Olmutinib inhibited the growth of a number of lung cancer cell lines [38]. At an IC_50_ of 10 nm., olmutinib inhibited EGFR (L858R/T790M) [39].

#### 4.5.3. Rociletinib

The 2,4-diaminopyrimidine-based compound rociletinib (CO1686) (Figure 2) has a reactive acrylamide group, an aminopyrimidine group, and a piperazine ring [40]. In an enzymatic experiment, rociletinib, a selective mutant EGFRL858R/T790M inhibitor, showed inadequate effectiveness against wild-type EGFR. When compared to erlotinib and afatinib in EGFR^T790M^, rociletinib showed dose-dependent tumor response and better activity, which was verified in transgenic mice experiments [40].

#### 4.5.4. Naquotinib

Naquotinib (Figure 2) is a 3-aminopyrazine-based drug that inhibits NSCLC cell lines with varied EGFR status (EGFR^L858R^, EGFR^del19^, EGFR^L858R^/^T790M^, and EGFR^del19/T790M^ mutations) with IC_50_ = 8–33 nM. compared to EGFR^WT^ (IC_50_ = 230 nM).

#### 4.5.5. Avitinib

Avitinib (Figure 2) is a structurally different pyrrolopyrimidine-based irreversible EGFR inhibitor compared to other pyrrolopyrimidine-based irreversible EGFR inhibitors. It suppressed mutant EGFR^L858R/T790M^ with an IC_50_ value of 0.18 nM. in a biochemical experiment, indicating around 43-fold more potency than EGFR^WT^ (IC_50_ = 7.68 nM). In phase I/II research, avitinib was tested in EGFR mutant patients who had progressed on first-line EGFR TKIs [41].

#### 4.5.6. Nazartinib

Nazartinib (Figure 2) is a novel epidermal growth factor receptor-tyrosine kinase inhibitor (EGFR-TKI) that is persistent and mutant-selective. It has been shown to be effective in treating patients with EGFR-mutated non-small cell carcinoma (NSCLC). [42]. With IC_50_ values of 4.18, 6.12, and 1.52 nM., respectively, Nazartinib, an aminobenzimidazole-based derivative, demonstrated encouraging efficacy against H1975, H3255, and HCC827 cells.

In both cell and enzymatic experiments, PF-06459988, with a pyrrolopyrimidine core structure, demonstrated significant potency and selectivity for the mutant EGFR^L858R/T790M^.

Despite the third-generation EGFR TKIs’ strong potency, the advancement of epigenetic mutation and acquired resistance has limited their usage in clinical concerns [33].

### 4.6. Fourth-Generation EGFR-TKIs: EAI001, EAI045, DDC4002, DDC-01-163 [43]

Recently, several fourth-generation allosteric EGFR inhibitors that bind to a site in the EGFR other than the PTK (protein tyrosine kinase) domain were reported. These inhibitors are ineffective against NSCLC with a mutant EGFR; when paired with an EGFR inhibitor such osimertinib or the monoclonal antibody cetuximab, they have synergistic anticancer benefits.

#### 4.6.1. EAI001

EAI001 (EGFR allosteric inhibitor-1) (Figure 2) was the first molecule to be found with such efficacy and selectivity for mutant EGFR (half maximal inhibitory concentration (at 1 mM ATP, IC_50_ = 0.024 µM for L858R/T790M, IC_50_ > 50 µM for wild-type EGFR).

#### 4.6.2. EAI045

EAI045 (Figure 2) was identified as an allosteric, non-ATP competitive inhibitor of mutant EGFR. EAI045 is the first inhibitor to be disclosed that can overcome both EGFR T790M and C797S mutations [44].

#### 4.6.3. Quinazoline-4-One-Based Derivatives

The replacement of the 2-aminothiazole amide group with the non-hydrolysable quinazoline-4-one core resulted in a number of quinazoline-4-one derivatives. Only TREA-0236 demonstrated EGFR^L858R/T790M/C797S^ inhibitory action in biochemical assays, with an IC_50_ of 5.3 µM.

#### 4.6.4. Aryl-4-Aminoquinazoline-Based Compounds

Based on virtual screening, 2-aryl-4-aminoquinazoline was identified as a fourth-generation triple mutant EGFR^del746−750/T790M/C797S^ inhibitor with potent kinase inhibitory enzymatic activity (IC_50_ = 149 nM) and 163-fold selectivity over EGFRWT.

#### 4.6.5. Tri-Substituted Imidazole Derivatives

Tri-substituted imidazoles with the aromatic alcohol group (phenol) or aliphatic alcohol were evaluated as EGFR^L858R/T790M/C797S^ mutant reversible inhibitors. With IC_50_ values less than 8 and 12 nM, these compounds showed potential inhibitory efficacy against mutant EGFR^L858R/T790M/C797S^ and EGFR^L858R/T790M^ without selectivity over EGFRWT (IC_50_ less than 0.5 nM [33].

#### 4.6.6. Amino Pyrazolopyrimidine-Based Compounds

Based on the structure of ibrutinib, 4-amino pyrazolopyrimidine derivatives are introduced as new mutant EGFR inhibitors [45]. This substance demonstrated highly effective inhibitory activity against the drug-resistant EGFR mutant L858R/T790M (IC_50_ less than 0.3 nM) and moderates antiproliferative activity against the EGFRBaF3 isogenic cell lines containing the C797S mutation (IC_50_ > 2 μM) in cell-based assays [33].

#### 4.6.7. Pyrimido-Pyrimidinone-Based Derivatives

Based on a random screening of 3000 compounds, a pyrimido-pyrimidinone derivative was found as a selective mutant EGFR^C797S^ inhibitor. This compound effectively inhibited mutant EGFR^C797S^ enzymatic activity (IC_50_ = 5.8 nM), as well as mutant EGFR^L858R/T790M/C797S^ and EGFR^19D/T790M/C797S^ phosphorylation in BaF3 cells (IC_50_ values of 0.51 and 0.32 µM, respectively) [46].

#### 4.6.8. Pyrido[3,4-d]Pyrimidine-Based Derivatives

New fourth-generation EGFR-TKIs, a novel class of pyrido[3,4-*d*]pyrimidine compounds, were designed, with this compound being the most promising, inhibiting the proliferation of HCC827 and H1975 cells with IC_50_ = 0.04 µM and displaying potent inhibitory activities against the mutants EGFRL858R (IC_50_ = 1.1 nM) and EGFRL858R/T790M/C797S (IC_50_ = 7.2 nM) [33].

#### 4.6.9. Purine-Based Derivatives

Novel 9-heterocyclyl substituted 9H-purines can act as tyrosine kinase inhibitors for the EGFR ^L858 R/T790 M/C797S^ mutant. The synthesized compounds D4, D9 (Figure 2), D11, and D12 show very powerful anti-proliferation and enzyme-based inhibition of EGFR^L858 R/T790 M/C797S^. The most effective drug, D9, specifically suppressed the proliferation of HCC827 and H1975 cells with IC_50_ values of 0.00088 µM and 0.20 µM, respectively, while also inhibiting the EGFR^L858 R/T790 M/C797S^ with an IC_50_ value of 18 nM. The IC_50_ value for D9’s inhibition of the EGFR^L858 R/T790 M/C797S^ was 18 nM. In addition, D9 dramatically reduced the phosphorylation of the EGFR, caused apoptosis, stopped the cell cycle at the G0/G1 stage, and decreased colony formation in the HCC827 cell line in a concentration-dependent manner [47].

### 4.7. Small Molecule Natural Products and Semi-Synthetic Derivatives

Small molecule natural products and semi-synthetic derivatives may up- or downregulate the expression of the human epidermal growth factor receptor (HER type), a prospective target in the regulation of signaling cascades implicated in excessive cellular proliferation [48]. Studies have demonstrated that some of the bioactive compounds in medicinal herbs overcome drug resistance to EGFR-TKIs and potentiate the therapeutic effects of EGFR-TKIs. These compounds include polyphenols, saponins, terpenoids, alkaloids, quinones, resins, and nucleosides.

It has been established that myricetin, kaempferol, and quercetin have anticancer properties via inhibiting receptor tyrosine kinases in medulloblastoma cells. Inhibiting the EGFR signaling-mediated pathways are the prospective pharmacological capabilities of kaempferol, silibinin, and apigenin. In the therapy of cancer, it has been discovered that caffeine (CA) targets receptor tyrosine kinases (RTK). RTKs include the epidermal growth factor receptor (EGFR), a cell-surface epidermal growth factor receptor. In breast cancer cells, caffeic acid prevents the phosphorylation of EGFR [49].

#### 4.7.1. Resveratrol

**Figure 3:**
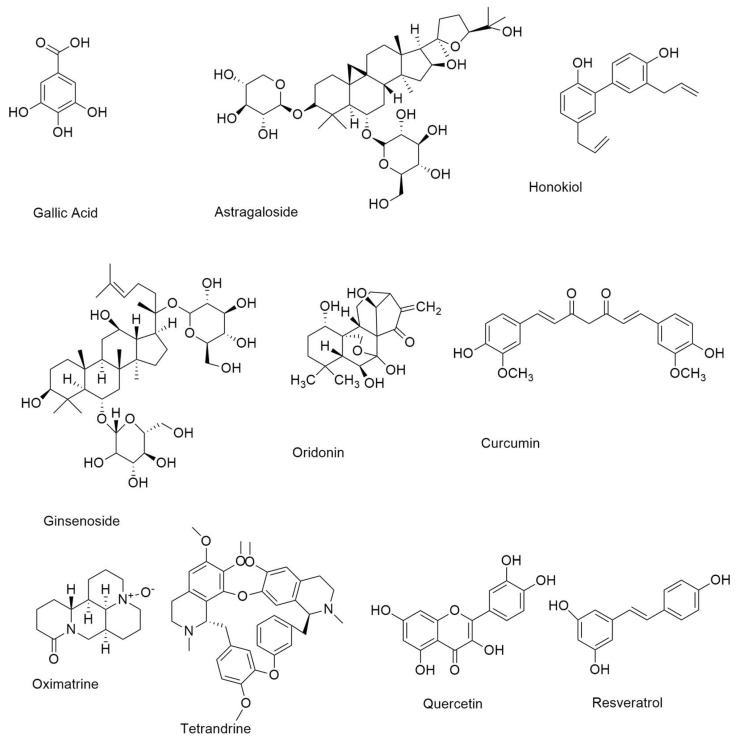
Small molecule natural compounds as EGFR inhibitors.

Resveratrol is a member of the stilbene family of polyphenolic chemicals. The anti-NSCLC properties of gefitinib are enhanced when combined with resveratrol. Resveratrol (Figure 3) may prevent cancer cells of prostate cancer by short-circuiting EGFR4-dependent autocrine loops [50].

#### 4.7.2. Quercetin

Quercetin (Figure 3), known as 3,3′,4′,5,7 pentahydroxy flavone (C_15_H_10_O_7_), is a glycoside and one of the most common polyphenolic flavonoids found in fruits and vegetables [49]. In a cirrhotic animal model, pancreatic tumor cells, and the EGFR-mediated signaling pathway, quercetin, and quercetin 3-*O*-glucoside, suppress EGFR expression.

#### 4.7.3. Gallic Acid

Gallic acid (Figure 3), a hydroxybenzoic acid found in a variety of plants and fruits, increases EGFR turnover, inhibits the Src-signal transducer and activator of transcription-3 (STAT3) signaling pathway, and causes apoptosis and cell cycle arrest in EGFR-TKI-resistant NSCLC. Gallic acid has been shown to inhibit EGFR, which in turn slows the progression of NSCLC in some cases [51].

#### 4.7.4. Curcumin

Curcumin is an isoflavonoid isolated from turmeric predominantly found in the south and southeast of Asia and has exhibited a broad spectrum of special biological functions such as neuroprotective effect, antioxidants, and anticancer. Curcumin (Figure 3), also known as diferuloylmethane, is an anti-NSCLC compound derived from the rhizome of *Curcuma longa*. It suppresses NSCLC cell growth and promotes death by raising caspase-3 activity and miR-192-5p expression. Curcumin also enhances gefitinib’s therapeutic effects. Ginseng roots, fruits, stems, and leaves contain ginsenosides, which are the major saponins [52].

#### 4.7.5. Ginsenoside

Ginsenoside (Figure 3) inhibited NSCLC cell proliferation by activating the tumor suppressor p53-binding protein-1. In NSCLC, ginsenosides enhance gefitinib anticancer activity [48].

#### 4.7.6. Astragaloside IV

Astragaloside IV (Figure 3) is a bioactive saponin discovered in the roots of *Astragalus membranaceus*, a Chinese medicinal herb. The main active saponins identified from *Paris polyphylla* are polyphyllin (PPI), PPII, and PPVII. PPI overcomes gefitinib resistance in NSCLC cells by lowering cell viability and triggering death via inhibiting the STAT3 signaling pathway and downregulating MALAT long non-coding RNA [53].

#### 4.7.7. Capilliposide

Capilliposide is a saponin found in *Lysimachia capillipes*, a Chinese medicinal herb that suppresses AKT activity, boosts gefitinib’s pro-apoptotic action, and reduces EGFR phosphorylation in gefitinib-resistant NSCLC cells [53].

#### 4.7.8. Cucurbitacins

Cucurbitacins are tetracyclic triterpenes identified from the *Cucurbitaceae* plant family, such as cucurbitacin B and D. Cucurbitacin B inhibits NSCLC development by causing cell cycle arrest in G2/M and mitochondrial death. Isodon plants *Rabdosia rubescens*, *Isodon japonicus* Hara, and *Isodon trichocarpus* produce oridonin, a diterpenoid chemical. Oridonin inhibits gefitinib-resistant NSCLC cells from growing.

*Sophora flavescens* produces the alkaloid matrine. By blocking the JAK/STAT3 signaling pathway, a combination of matrine and afatinib, an EGFR-TKI, increases the growth inhibitory effects of NSCLC cells. Shikonin is a naphthoquinone chemical derived from the root of Lithospermum erythrorhizon, a Chinese medicinal herb. Shikonin causes apoptosis and cytotoxicity in NSCLC cells through increasing ROS generation and cytotoxicity [53].

#### 4.7.9. Oridonin

Oridonin (Figure 3), a bioactive diterpenoid component, has been shown to suppress cancer cell proliferation via blocking the EGFR signaling pathway.[54]. With the administration of oridonin, the expression of EGFR downstream proteins such as GRB2, Ras, Raf, ERK, and JNK was also reduced [55].

#### 4.7.10. Honokiol

Honokiol (Figure 3), a neolignane derivative extracted from *Magnolia grandiflora* bark and leaves, has a number of anti-cancer activities, while being quite safe [56]. According to research on the EGFR downstream signaling pathway, honokiol can downregulate the PI3K/Akt/mTOR system by reducing Akt phosphorylation and raising PTEN expression, [57].

#### 4.7.11. Oxymatrine

Oxymatrine (Figure 3), a bioactive alkaloid derived from the *Sophora flavescens* Aiton plant, has long been used to treat cancer, chronic hepatitis, and inflammatory illnesses. Oxymatrine has been shown to suppress cell proliferation and induce apoptosis in cancer cells, including gall bladder carcinoma, colorectal cancer, and leukemia [58]. To conduct its cytotoxicity against cancer cells, oxymatrine can control numerous oncogenic signaling pathways, including the Akt, EGFR, and NF-κβ cascades [59].

#### 4.7.12. Tetrandrine

In China, the *bis*-benzyl isoquinoline alkaloid tetrandrine (Figure 3) has been used to treat cancer, inflammation, asthma, and hypertension [60]. Tetrandrine blocks EGFR phosphorylation and downstream signaling pathways such as PI3K/Akt and MAPK [61].

### 4.8. Combination of Natural and Synthetic Compounds

Synthetic compounds and natural compounds have a synergistic effect. Natural compounds have as good an effect as EGFR-TKIs on their own, but the dose of natural compounds can be lowered when combined with synthetic molecules. With EGFR-TKIs, it is necessary to combine natural and synthetic compounds [52].

## 5. Lung Cancer

**Figure 4:**
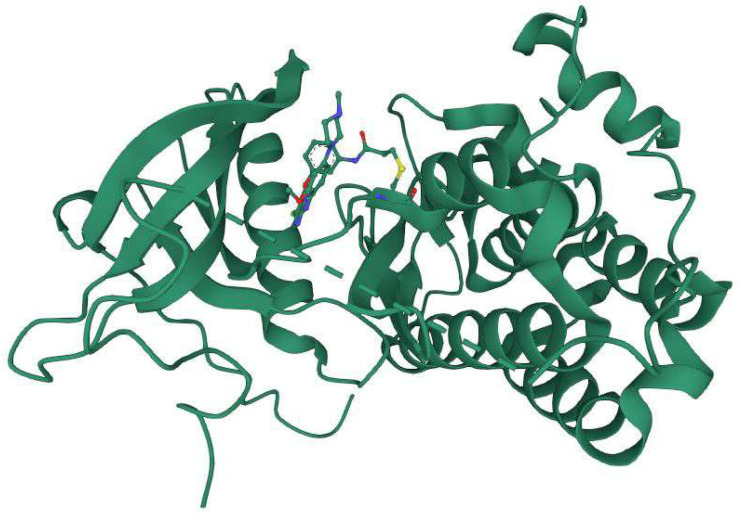
Co-crystallized structure of EGFR T790M with a pyrimidine derivative WZ4002 (PDB ID: 3IKA) (Image courtesy of RSCB PDB: https://www.rcsb.org, accessed on 31 December 2022) [64].

Lung cancer is the most common cancer and kills people all over the world. It is divided into two major histologic types: Small-cell lung cancer and non-small-cell lung cancer. Epidermal growth factor receptor is expressed in more than 60% of NSCLC tumors (EGFR) [62]. The characterization of NSCLCs at the molecular level has given useful information for diagnosis, prognosis, and treatment. EGFR can be activated by overexpression or by ligand-dependent or ligand-independent mechanisms [63]. In 2004, three groups compared the tumors of patients who reacted to gefitinib versus those who did not. These researchers discovered that the majority of responders had mutations in the EGFR kinase domain, but the non-responders did not. The most typical mutations discovered by these researchers were (i) deletion of five exon-19 residues just before the C-helix and (ii) an arginine for leucine (L858R) substitution in exon-21 of the activation zone. Patients who responded to erlotinib also possessed these EGFR mutations. More than 200 EGFR mutations have been discovered in NSCLC. Gefitinib and erlotinib are reversible EGFR inhibitors based on quinazoline that are used to treat NSCLC with EGFR exon-19 deletions and the exon-21 L858R mutation. With a median length of 10–13 months, almost all NSCLC patients with EGFR-activating mutations acquire resistance to these medicines. The development of the exon 20 T790M gatekeeper mutation is the most common resistance mechanism. Afatinib, like gefitinib and erlotinib, is a quinazoline derivative that inhibits the activated L858R gatekeeper mutant by creating a covalent connection with EGFR C797. There are another four FDA-approved drugs: dacomitinib (which targets mutant EGFR in lung cancer), neratinib (which targets ErbB2 in HER2-positive lung cancer), osimertinib (which targets EGFR T970M mutants in NSCLC), and ibrutinib (which targets BTK in mantle cell lymphoma, chronic lymphocytic leukemia, marginal zone lymphoma, and chronic graft vs. host. Osimertinib is a covalent EGFR TKI that is a mono-anilino-pyrimidine molecule. Osimertinib showed strong action against a variety of EGFR mutations in EGFR-recombinant enzyme assays (L858R, L858R/T790M, exon 19 deletions, and exon 19 deletion/T790M) [4]. A representative example of the drug–protein complex with the T790M mutant is shown in Figure 4. Here, a mutant-selective EGFR TKI (a pyrimidine derivative WZ4002) is covalently bound to the crystal structure of the EGFR 8696-1022 T790M mutant (PDB ID: 3IKA). This class of compound co-crystallizes with EGFR T790M approximately 30–100 times more efficiently than wild-type EGFR and subsequently demonstrated (in vitro) 30–100 times more potency than wild-type EGFR [34].

Naquotinib (ASP8273), another small molecule heterocycle, having comparable activity with Osimertinib, was developed by Astellas Inc. and showed promise in the in vitro and in vivo studies against *EGFR* T790M+. However, in the U.S. phase I clinical trial, it failed to reach the maximum tolerated dose (MTD) [65]. Later, a phase III randomized study of naquotinib contrasted with erlotinib or gefitinib in patients with advanced stage IIIB/IV NSCLC naquotinib demonstrated inferior to even first-generation TKIs. The grade 3 hyponatremia of naquotinib was 20.4% compared to less than 1% for erlotinib/gefitinib. Moreover, the percentage of grade 3 diarrhea was more than double in the naquotinib cohort compared to erlotinib/gefitinib [66]. JBJ-09-063, a mutant-specific allosteric EGFR inhibitor (inhibitor that binds to the allosteric site of the protein) has recently been reported [67]. This small heterocyclic molecule exhibited excellent IC50 values for EGFRL858R, EGFRL858R/T790M, EGFRL858R/T790M/C797S, and EGFRLT/L747S (0.147 nM, 0.063 nM, 0.083 nM, and 0.396 nM, respectively).

It is worth mentioning that drug–protein interactions through molecular docking studies may produce erroneous outcomes because of the structural rigidity of the biomolecular targets, which is in contrast to reality. To avoid or minimize this error, molecular dynamics (MD) simulations can be employed to illustrate the molecular properties of the biomolecular targets, protein–ligand interactions, and related conformational changes that may happen in response to some perturbations. In the MD simulation study, both the perturbed and unperturbed environments are considered to determine an optimized result. Sanders et al. [68] evaluated the binding interactions between the extracellular domain of EGFR with seven ligands through MD simulation studies [68]. While simple biophysical analyses can describe the interactions at the molecular levels (intermolecular interactions), the MD simulation explains the interactions at the atomic levels through atom-by-atom resolution and dynamical behavior on a nanosecond timescale. Exploring the theoretical free energy methods combined with conventional MD simulations, the authors [67] reported the binding affinities of each ligand through residue-residue interactions.

## 6. Breast Cancer

With over a million cases diagnosed each year, breast cancer is a primary cause of morbidity and mortality globally. In 1987, Slamon et al. discovered that the HER-2/neu oncogene is amplified and overexpressed in 20–25% of breast tumors. Amplification of HER2 leads to homo- and heterodimerization with other members of the ErbB family, which promotes cell proliferation, survival, and the angiogenic pathway. Lapatinib ditosylate is a reversible, small-molecule tyrosine kinase dual inhibitor of EGFR and HER2. It belongs to the quinazoline family and has a 4-anilinoquinazoline core. Lapatinib binds to an inactive-like conformation of EGFR and has a slower inhibitor dissociation rate than erlotinib and gefitinib, with estimated Ki-app values of 3 nM and 13 nM against EGFR and ErbB2, respectively. This was linked to a prolonged downregulation of EGFR phosphorylation in EGFR-overexpressing HN5 cells. Lapatinib caused considerable survivin protein downregulation and associated apoptosis in HER2-overexpressing cell lines. Lapatinib not only inhibited HER2 phosphorylation, but also decreased HER2 receptor ubiquitination and increased the accumulation of inactive HER2 receptors at the cell surface, according to recent in vitro work using HER2-overexpressing breast cancer cell lines (SKBR3 and MCF7-HER2) and correlation with in vivo data using BT474 xenografts [19].

## 7. Colorectal Cancer

For metastatic colorectal cancer (CRC), drugs targeting the epidermal growth factor receptor (EGFR), such as cetuximab and panitumumab, have been recommended, but patients with KRAS mutations are unresponsive to them and have no other options. Adenomatous polyposis coli (APC) mutations, which occur in 90% of human CRCs, enhance the levels of β-catenin, EGFR, and RAS, particularly mutant KRAS, in CRC patient tissues. KYA1797K, a newly discovered small molecule that activates GSK3 to destroy both β-catenin and Ras, and its capacity to reduce cetuximab resistance in KRAS-mutated CRC cells. Small molecules that degrade both β-catenin and RAS via EGFR transcriptional repression could be a potential therapy for CRC patients with abnormally activated Wnt/β-catenin and EGFR-RAS pathways due to an increase in β-catenin, EGFR, and RAS, as well as their activation by pathologically important APC, KRAS, and EGFR mutations. KYA1797K suppressed the proliferation of several CRC cells in a dose-dependent manner, regardless of their KRAS mutation status. EGFR is likewise overexpressed in human CRC and plays a role in tumorigenesis synergy. Inhibition of the Wnt/β-catenin and EGFR-RAS-ERK pathways, particularly by lowering the amounts of proteins increased in CRC, could be an ideal treatment for human CRC. Small molecules that degrade both β-catenin and RAS while reducing EGFR transcription could thus be promising therapeutic candidates for the treatment of CRC, particularly CRCs resistant to EGFR-targeting treatments due to KRAS mutation [69].

## 8. Brain Tumor

The most common malignant primary brain tumor, glioblastoma (GBM), has a poor prognosis despite surgery, radiation, and chemotherapy. EGFR mutations are seen in about 45 % of GBM cases. Patients with GBM usually have abnormalities in platelet-derived growth factor receptor (PDGFRA) and mutations in the vascular endothelial growth factor (VEGF)/VEGF receptor (VEGFR) in addition to EGFR dysregulation [70]. Bay846 is a novel small molecule inhibitor that binds permanently to the EGFR and HER2 tyrosine kinase domains. Bay846 is a Bayer Healthcare-developed-and-provided irreversible dual EGFR/HER2 inhibitor (Berlin, Germany). An MTT experiment was used to compare Bay846’s capacity to suppress cell proliferation to that of lapatinib. Bay846 significantly reduces active, phosphorylated EGFR levels, suppresses tumor cell proliferation, causes tumor cell lysis, and has potent anti-tumor activity, resulting in a high frequency of tumor size regressions. Bay846 outperforms lapatinib, a reversible EGFR, and HER2 inhibitor. These findings back up the idea that irreversible EGFR tyrosine kinase inhibitors will be more effective than reversible inhibitors. Bay846 is a highly effective and powerful EGFR inhibitor [71].

## 9. Renal Cell Cancer

Renal cancer carcinoma accounts for approximately 3% of all adult cancers. RCC was the 10th most prevalent cause of cancer mortality in men in the United States in 2003, with 31,000 patients diagnosed with the disease and 12,000 deaths. EGFR is expressed in 50–90% of RCCs. Phase II trials of three TKIs in renal cell cancer (sorafenib, sunitinib, and AG 013736) show that the drugs are effective in treating second-line RCC, with partial responses in up to 40% of patients. Sorafenib has been shown to be effective as a second-line single agent in a randomized phase III trial. Vatalanib inhibits the growth of a murine renal carcinoma model in vivo [7].

## 10. Head and Neck Squamous Cell Carcinoma

Squamous cell carcinomas (SCC) of the head and neck are cancers that develop mostly in the mucosa of the oral cavity, sinuses, oropharynx, hypopharynx, and larynx [72]. EGFR is overexpressed in over 90% of head and neck cancers, and that results in patients living shorter lives. As a single therapy or in combination with radiation treatment, gefitinib has shown limited clinical efficacy, with response rates of 10–15% in HNSCC patients. In a mouse model, osimertinib has exceptional effectiveness against tumors overexpressing HER2 (ErbB2) [73].

## 11. Bone Tumor

Chordoma is a rare primary malignant bone tumor that originates mostly in the bones of the base of the skull and shows notochordal differentiation. Anecdotal reports on the responsiveness of chordoma to epidermal growth factor receptor (EGFR) inhibitors are promising. Six EGFR inhibitors, erlotinib, gefitinib, sapitinib, afatinib, poziotinib, and lapatinib, were purchased for screening. Erlotinib, gefitinib, and sapitinib, three of the four reversible agents tested, were highly powerful, with effects in the nanomolar range in the four ‘responsive’ cell lines. Lapatinib, on the other hand, was highly effective on UM-Chor1 (EC_50_ = 320 nM and 98% MI), but only moderately effective on MUG-Chor1 (EC_50_ ≥ 1 µM ≤ 3 µM) and had no effect on the other cell lines (EC_50_ ≥ 3 µM). The first of the two irreversible EGFR/ERBB inhibitors, afatinib, showed a strong death effect on UM-Chor1 (EC_50_ = 26 nM and 89% MI). Sapitinib, a ‘quinazoline small’ molecule, showed the most promising results, with EC_50_ concentrations in the nanomolar range in the four ‘responsive’ lines, which were comparable to those shown in non-small cell lung cancer (NSCLC) and head and neck cancer cell lines designated as EGFR-sensitive. Gefitinib and erlotinib, two FDA-approved ‘quinazoline small’ compounds, were the other active molecules [74].

## 12. Cutaneous Squamous Cell Carcinoma in Skin (SCC)

Skin cancer is the most common type of cancer in humans [75]. EGFR is overexpressed in psoriatic skin, not only in the basal epidermal layer, but also in other layers [76]. There are a few therapeutic options for individuals with advanced SCC, such as cisplatin-based combination chemotherapy, which has a good response rate but a high frequency of side effects. EGFR overexpression is found in up to 43% of advanced SCC. In 15% of patients, gefitinib produces only a partial response. Anti-EGFR antibodies and tyrosine kinase inhibitors may be useful in the treatment of SCC [75].

## 13. Conclusions

The applications centered on EGFR are the most promising and advanced used at the clinical level among recent developments in molecular targeted cancer therapy [52]. Approximately 70% of patients with a sensitizing mutation respond to EGFR inhibitor therapy; however, after an average of 10–11 months, most individuals acquire resistance to these drugs [77]. According to the National Cancer Institute (NCI), EGFR protein is found in specific cell types and is involved in the cell signaling pathways that control cellular division and survival. Due to mutation in the *EGFR* gene, an excess amount of EGFR protein is generated in some types of malignant cells, which causes a rapid cell division, and ultimately one or more malignant tumors result. EGFR inhibitors can potentially dock this protein, and these inhibitors can be used in the treatment of certain types of cancers related to overexpression of the *EGFR* gene [78]. This article overviews a wide range of small molecules, both synthetic and natural, that are capable of inhibiting EGFR. Particular attention was paid to the nature of the small molecule and its mechanisms of action. The use of EGFR-TK inhibitory action revolutionizes cancer treatment. TKIs, which target EGFR, have proven to be highly effective in treating some of the most common and difficult cancers, including lung, colon, breast, head and neck cancers. So far, fourteen small-molecule EGFR inhibitors have been certified for the treatment of various malignancies. According to currently known research, combining an allosteric EGFR inhibitor with chemo- or immunotherapy is far more successful than either treatment alone. Scientists who are involved in this study may pay attention to this thought.

## Figures and Tables

**Table 1:** The synonym and discovery of four members of the EGFR family.

Receptor	Synonym	Discovery	References
EGFR	erbB1, HER	Carpenter et al. (1975) and Ullrich et al. (1984)	[6]
ErbB2	HER2, neu	Stern et al. (1986); Bargmann and Weinberg (1988)	[6]
ErbB3	HER3	Plowman et al. (1990)	[6]
ErbB4	HER4	Plowman et al. (1993)	[6]

**Table 2:** Classification of EGFR TKIs based on the nature of inhibition of EGFR [10].

Reversible Inhibitors	Irreversible Inhibitor
Brigatinib	Afatinib
Erlotinib	Neratinib
Gefitinib	Pyrotinib
Icotinib	Almonertinib
Lapatinib	Olmutinib
Simotinib	Osimertinib
Vandetanib	Dacomitinib

**Table 3:** Properties of selected orally effective small-molecule EGFR family inhibitors [4].

Name (Trade Name)	Targets	FDA-Approved Indications or Clinical Trial Study (Year)
Gefitinib	EGFR	NSCLC (2003)
Erlotinib	EGFR	NSCLC (2004) and pancreatic cancer (2005)
Afatinib	ErbB1/2/4	NSCLC (2013)
Osimertinib	EGFR	NSCLC (2015)
Dacomitinib	Pan-HER	NSCLC (2018)
Lapatinib	EGFR/ErbB2	Breast cancer (2007)
Neratinib	ErbB2/HER2	Breast cancer (2015)
Avitinib	EGFR	Phase I and II clinical trials for NSCLC
Olmatinib	EGFR	Phase II clinical trials for NSCLC
Pelitinib	EGFR	Phase I clinical trials for NSCLC and colorectal cancer
Pyrotinib	EGFR, ErbB2	Gastric, breast, NSCLC
Brigatinib	EGFR	NSCLC, leukemia
Vandetinib	EGFR	NSCLC, thyroid, liver, breast, CRC
Icotinib	EGFR	Brain, NSCLC, pancreas, head and neck
